# Defeating re-emerging Alkhurma hemorrhagic fever virus outbreak in Saudi Arabia and worldwide

**DOI:** 10.1371/journal.pntd.0006707

**Published:** 2018-09-27

**Authors:** Ernest Tambo, Ashraf G. El-Dessouky

**Affiliations:** 1 Biochemistry and Molecular Biology Unit, Public Health Pests Laboratory, Jeddah Governorate, Jeddah, Saudi Arabia; 2 Microbiology and Parasitology Unit, Public Health Pests Laboratory, Jeddah Governorate, Jeddah, Saudi Arabia; 3 Biochemical Genetic Unit, Medical Genetics Center, Faculty of Medicine, Ain Shams University, Cairo, Egypt; Kwame Nkrumah University of Science and Technology, GHANA

Sporadic incidence and prevalence of Alkhurma hemorrhagic fever virus (AHFV) (family Flaviviridae; genus *Flavivirus*) in Saudi Arabia (KSA) is profiled periodically. The virus, which is related to tick-borne encephalitis (TBE) complex and is genetically closely related to Kyasanur Forest disease virus (KFDV), was primarily identified in KSA in November and December 1995 based on isolation from the blood of 6 male butchers aged 24 to 39 years old and residents in the Jeddah province, of whom 4 recovered completely [1,2]. Since then, new outbreaks with sporadic incidences have been reported in KSA, and subsequent cases of AHF have been documented among tourists in Egypt and Djibouti, extending to India, Europe, and beyond, suggesting that AHFV infections’ geographic distribution is underreported [3,4,5].

According to the Saudi Ministry of Health statistical yearbook for the year 2016 (1437 H), 38 new infection cases were reported in 2016 from the Makkah and Najran provinces. Twenty-four of the infections affected those aged 15 to <45 followed by 13 cases among those aged ≥45 [6]; meanwhile, a low incidence rate was documented among Saudi citizens compared to foreigners/expatriate workers, with the highest infection rate during the months of March, July, and September yearly [7]. The disease infection pattern revealed that the reported cases were 58, 59, 70, 60, and 38 for 2012, 2013, 2014, 2015, and 2016, respectively, per 100,000 population in KSA [7].

AHFV cases have been epidemiologically linked to direct and indirect exposure (to infected blood and/or organs of slaughtered animals and consumption of infected raw milk. Assumptions of infection transmission via arthropod bites, including *Hyalomma* spp. ticks and migratory birds and/or small and/or larger mammals (e.g., cattle, buffaloes, camels), have been reported in the literature [8,9,10] even though this assumption was not supported by other previous studies’ reports [11]. A retrospective analysis of AHFV laboratory-confirmed cases for 3 consecutive years (2009–2011) reported that the most common signs and symptoms were fever (reported by about 96% of all cases, with more than 90% having body temperatures above 37.0° C), malaise (about 60%), and chills (about 40%) [8,10]. Clinical manifestations also included pain disorders and headache (60%), myalgia (44%), retro-orbital pain (16%), gastrointestinal symptoms (99%), anorexia (62%), nausea (60%) and vomiting (53%), and diarrhea (12%). Whereas less than 10% of the patients reported any symptoms of the central nervous system, the most common symptoms were disorientation (5.4%), hallucination (4.3%), and convulsion (4.3%) [8,9,10]. The most common hemorrhagic manifestations were purpura and epitasis (5%), whereas the least was menorrhagia (2.2%), and the overall case fatality rate was 0.4% [12]. Moreover, leukopenia, thrombocytopenia, and elevated levels of liver enzymes were reported in infected patients [3].

AHFV, tick-borne zoonotic disease outbreak, has been reported on shepherds, cattle owners, butchers, and sometimes pilgrims who slaughter sacrificial animals and transmitted via contact with infected animals or tissues, including sheep, goats, and camels [8,12]. In contrast, no serological evidence or virus isolation efforts were reported for or from the incriminated animal hosts, reservoir, and arthropod vector, thus pointing to a lack of a robust and integrated AHFV laboratory and epidemiological surveillance system in mapping ecological hotspots in KSA and other affected regions [11,12,13]. Thus, Alkhurma hemorrhagic fever epidemiology is not fully understood, and knowledge gaps related to its transmission route(s), key vector(s), host reservoir(s), population-based virulence, and clinical consequences demand much in depth research. The role of tick(s) as a disease vector is based on the assumption that AHFV is a serologically and genetically variant serotype closely related to KFDV—having 89% sequence homology, suggesting a common ancestral origin [13]—and KFDV documented transmission is primarily through tick bites [14,15]. Remi and colleagues reported the detection of AHFV RNA in one *Ornithodoros savignyi* tick from KSA by RNA product reverse transcription (RT)-PCR) sequencing in 2007 [16], with subsequent reports in Djibouti [17]. Such findings need further validation with large populations of human and/or tick surveillance and confirmatory evidence through virus characterization, serological and virus RNA genomic detection, and mapping of potential risk factors to reveal hemolymph type, source of infection, and role in viral transmission. Unfortunately, robust tick and tick-borne disease surveillance and mapping—including highly pathogenic AHFV genetic diversity, molecular characterization, and phylogenetics analysis—is lacking, and the poor understanding of the disease cycle challenged evidence decision-making support and targeted interventions in KSA. Meanwhile, AHFV human-to-human transmission and geographical distribution have not yet been fully elucidated in KSA and worldwide [3,13,15,18].

In the absence of effective vaccines and drugs, only palliative clinical case management is presently available. Consequently, integrating a “One Health” approach in increasing AHFV preparedness, response interventions capacity building, and outbreak surge contingency capacity is crucial in fostering community-based approaches and strategies implementation against reservoir-linked imported AHFV and other emerging epidemics from endemic settings into new areas [18,19], including the following:

1. Re-enforcing integrated public health laboratory surveillance on existing invasive pathogenic threats and emerging pandemics for evidence-based preparedness and response strategies, including community awareness and health education as well as capacity building and training of public–private and community health professionals in averting AHFV transmission dynamics and potential public health burden
2. Promoting the implementation of a local, regional, and global “One Health” approach policy and framework to understanding human–animal–environment interplay-related tick-borne diseases and monitor the emergence and spread impact of AHKV outbreaks and coinfections
3. Strengthening contextual hospital and/or clinical- and laboratory-based infection prevention and control best practices and strengthening precautionary measures against AHKV and other emerging pathogens that can cause unspecific, unusual, or unknown clinical signs and symptoms
4. Leveraging cutting-edge cloud-sourcing, social media, and internet-based network advancements to improve early-warning alerts, risk assessment, and mapping for appropriate and timely treatment and contextual response solutions
5. Understanding the key drivers or risk factors as well as host immune status and seroconversion mechanisms that could be important due to tick-borne flavivirus diseases, antibody cross-reactivity, and individual genetics and ecology
6. Investing in research and development (R&D) for point-of-care and field-adaptable serologic and molecular diagnostic tools for early detection, surveillance investigation tools, and safe and effective vaccines and drugs for (human and animal) effective case management
7. Bolstering AHKV risk-mapping quality data gathering for temporal–spatial modeling and prognostic and/or early warning alerts of potential AHKV epidemics and/or pandemics threat toward guided technical assistance and experts’ recommendations on timely community risk communication strategies. This is critical for strengthening a “One Heath” community of practice implementation for Saudi health transformation 2020 and Saudi vision 2030, leading to upholding global health security delivery and socioeconomic diversification benefits
